# Genomic analyses of the *Chlamydia trachomatis* core genome show an association between chromosomal genome, plasmid type and disease

**DOI:** 10.1186/s12864-018-4522-3

**Published:** 2018-02-09

**Authors:** Bart Versteeg, Sylvia M. Bruisten, Yvonne Pannekoek, Keith A. Jolley, Martin C. J. Maiden, Arie van der Ende, Odile B. Harrison

**Affiliations:** 1 Public Health Laboratory, Department of Infectious Diseases, Public Health Service Amsterdam, Amsterdam, the Netherlands; 2 Amsterdam Infection & Immunity Institute, Academic Medical Center, University of Amsterdam, Amsterdam, the Netherlands; 3 Department of Medical Microbiology, Academic Medical Center, Amsterdam, the Netherlands; 4 Peter Medawar building, Department of Zoology, University of Oxford, Oxford, UK

**Keywords:** Genome sequencing, *Chlamydia trachomatis*, Genomics, Plasmid

## Abstract

**Background:**

*Chlamydia trachomatis* (Ct) plasmid has been shown to encode genes essential for infection. We evaluated the population structure of Ct using whole-genome sequence data (WGS). In particular, the relationship between the Ct genome, plasmid and disease was investigated.

**Results:**

WGS data from 157 Ct isolates deposited in the Chlamydiales pubMLST database (http://pubMLST.org/chlamydiales/) were annotated with 902 genes including the core and accessory genome. Plasmid associated genes were annotated and a plasmid MLST scheme was defined allowing plasmid sequence types to be determined. Plasmid allelic variation was investigated. Phylogenetic relationships were examined using the Genome Comparator tool available in pubMLST. Phylogenetic analyses identified four distinct Ct core genome clusters and six plasmid clusters, with a strong association between the chromosomal genotype and plasmid. This in turn was linked to *ompA* genovars and disease phenotype. Horizontal genetic transfer of plasmids was observed for three urogenital-associated isolates, which possessed plasmids more commonly found in isolates resulting from ocular infections. The *pgp3* gene was identified as the most polymorphic plasmid gene and *pgp4* was the most conserved.

**Conclusion:**

A strong association between chromosomal genome, plasmid type and disease was observed, consistent with previous studies. This suggests co-evolution of the Ct chromosome and their plasmids, but we confirmed that plasmid transfer can occur between isolates. These data provide a better understanding of the genetic diversity occurring across the Ct genome in association with the plasmid content.

**Electronic supplementary material:**

The online version of this article (10.1186/s12864-018-4522-3) contains supplementary material, which is available to authorized users.

## Background

*Chlamydia trachomatis* (Ct) is responsible for the majority of bacterial sexually transmitted infections worldwide [1]. In addition, ocular Ct infections (trachoma) are the world’s leading cause of preventable blindness [2, 3]. Although there are few documented reports of antibiotic resistance in Ct and infections can be easily treated, the persistent rates of Ct globally, makes this infection an important public health priority.

Ct isolates can be grouped into 15 main genovars based on sequence data of *ompA,* the gene encoding the major outer membrane protein [4–6]. Specific genovars have been strongly associated with distinct disease pathologies: genovars A-C are associated with conjunctival epithelia; genovars D-K with urogenital, pharyngeal and anorectal epithelia; and genovars L1-L3 with submucosal connective tissue layers resulting in dissemination to locoregional lymph nodes and lymphogranuloma venereum (LGV) [7]. Ct clonal groups identified through different multilocus sequence typing (MLST) schemes based on 7 housekeeping genes as well as phylogenetic analyses of whole-genome sequence (WGS) data [8–11], have also shown an association between Ct strains and tissue tropism.

Ct isolates possess multiple copies of highly conserved small 7.5-kb plasmids containing both non-coding RNA of which the function is unknown, and 8 open reading frames (ORFs), designated *pgp1* to *pgp8* [12, 13]. The plasmid of Ct has been shown to encode genes essential for infection and transmission, consistent with the rare occurrence of plasmid-deficient clinical isolates [14–16]. The essential role of the plasmid in virulence and inflammatory responses was further demonstrated using animal models, where plasmid-deficient Ct strains or those with mutated plasmids were found to exhibit reduced pathology and decreased inflammatory responses [17–23]. All plasmid-borne genes are transcribed and at least one protein (*pgp3*) is known to be expressed. Putative functions have been assigned to some of the plasmid genes, based on homology to known proteins [13, 24–26], with *pgp1* exhibiting homology to a *DnaB* like helicase, *pgp5* to a partitioning protein which may regulate expression of a set of chromosomal genes, and *pgp7* and *− 8* identified as integrase/recombinase homologues. In contrast, *pgp*2, and *pgp6* genes are unique to the chlamydia genus. The product of *pgp*3 is secreted into the host cell cytosol and is the most studied plasmid gene, both as a serological marker for past infection and as virulence factor, as it was demonstrated to play an important role in the induction of inflammatory responses [21, 27, 28]. Finally, *pgp4* is a transcriptional regulator of *pgp3* and of some chromosomal genes that are likely to be important for chlamydial virulence [25]. The *pgp7* gene is not essential for plasmid maintenance as it was found to be interrupted in naturally occurring Ct strains resulting in the emergence and rapid spread of a new Ct variant [29]. This new variant originated in Sweden in 2006 and had a 377 bp deletion in the *pgp7* gene that prevented detection of infections using plasmid based PCR diagnostics targeting this gene [13, 29]. The potential of spread and emergence of new strains due to genetic variation indicates the need for more comprehensive studies to better understand the Ct genetic population structure.

Although it is clear that the plasmid plays an important role in the pathogenesis of Ct infection, limited data is available on its genetic diversity and whether distinct plasmid types are associated with different tissue tropisms and pathologies. This study set out to characterise the population structure of Ct in association with the plasmid using WGS data from 157 isolates available in the *Chlamydiales* pubMLST database (http://pubMLST.org/chlamydiales/). A better understanding of genetic diversity across the Ct genome in association with plasmid content may elucidate Ct epidemiology, ultimately reducing the burden of infection.

## Methods

### Ct isolate collections and WGS methods

Whole-genome sequence (WGS) data from 157 Ct isolates were analysed (Additional file 1: Table S1). WGS data were obtained from published isolate collections for which plasmid sequence data were also available [9, 26, 30–32]. Short reads were obtained from the European Nucleotide Archive (ENA) and assembled de novo using VELVET in combination with VELVETOPTIMISER as previously described [33]. The resulting contigs were uploaded to the Bacterial Isolate Genome Sequence (BIGSdb) genomics platform hosted on www.pubMLST.org/chlamydiales.

The Chlamydiales pubMLST platform consists of two types of database: i) a sequence definition database that contains sequences of known alleles for loci as well as allelic profiles for specific schemes such as MLST; and, ii) an isolate database that contains isolate provenance and other metadata along with nucleotide sequences associated with that isolate [34]. Sequence definitions have been established for 902 protein-encoding genes, annotated with the CHLAM prefix, and the majority of these have been organised into schemes dependent on function (Additional file 2: Table S2). Chromosomal genes were defined using the annotated genome from Ct strain D/UW-3/CX (accession number NC_000117, [35]).

The BIGSdb software includes ‘autotagger’ and “autodefiner” tools which scan deposited WGS against defined loci identifying alleles ≥98% sequence identity. This process runs in the background and automatically updates isolate records with specific allele numbers, marking regions on assembled contigs for any of the defined loci. Loci with sequence identity < 98% are manually checked and curated.

*OmpA,* which encodes the major outer membrane protein, was annotated in WGS data as CHLAM0681. Nucleotide sequence data from CHLAM0681 was extracted from all WGS. Bionumerics software (version 7.5, Applied Maths, Sint-Martens-Latem, Belgium) was used to import all extracted *ompA* sequences to a local offline reference database of *ompA* sequences that had been described in previous Ct studies [10]. Based on sequence similarity of the *ompA* variable domains 1 and 2, *ompA* genovars were assigned to all isolates. Genovars A-C were considered to be ocular isolates, genovars D-K, urogenital isolates and genovar L, LGV isolates. Genovar B is known to cause both urogenital and ocular infections.

### Phylogenetic analyses

Relationships among isolates were established using the Genome Comparator tool implemented within the *Chlamydiales* pubMLST database [34]. Genome Comparator compares groups of shared genes among isolates with any number of loci predefined in the *Chlamydiales* database or a reference genome. For each locus, allele sequences, designated by integers, are compared and used to generate a distance matrix that is based on the number of variable loci across the genome generating a wgMLST profile. Genome Comparator output provides lists of loci that are: i) identical, ii) variable, iii) missing, or incomplete between data sets, rapidly resolving bacterial population structures and relationships, and identifying loci that belong to the core of a particular data set [33].

Using the Genome Comparator tool, all chromosomal genes identified in the automated annotation process were compared. The set of 888 genes shared between 95% of all Ct isolates was referred to as the ‘core genome’. Varying the stringency of the core genome threshold did not have a significant impact on the results in this study, as a threshold of 90% resulted in a core genome of 889 genes while a threshold of 97.5% resulted in a core genome of 886 genes. Genome Comparator was used to compare the core genome among all isolates and to generate a distance matrix based on the number of variable loci. In addition, this was used to compare previously identified plasmid genes among all isolates. The generated distance matrix for the core genome and plasmid genes were further analysed using the NeighborNet algorithm in SplitsTree version 4.14, to investigate the phylogenetic clustering of Ct isolates according to both their core genome and plasmid loci [36]. Maximum Likelihood phylogenetic trees were also generated from concatenated aligned nucleotide sequence data derived from both core genome and plasmid loci using PhyML [37] and, the HKY85 model with 100 bootstraps. In addition, to each isolate a unique ID, the corresponding *ompA* genovar and the plasmid sequence type (pST) were linked to each isolate. ClonalFrameML [38] was also used with default parameters to take into account recombination events.

### Ct plasmid

Sequences from the plasmid belonging to Ct strain D/SotonD6 were retrieved from plasmid pSotonD6 (HE603231) and designated as CHLAM0895 through to CHLAM0902 encoding the genes *pgp1* to *pgp8*. Using Blast, all WGS sequence data deposited in pubMLST were annotated for these loci as described previously [34, 39, 40].

The eight Ct plasmid genes were grouped into a plasmid MLST typing scheme and plasmid sequence types (pSTs) were assigned based on identified allele variants for isolates with sequence data on all eight plasmid genes.

The number of polymorphic sites per plasmid gene, was assessed using the locus explorer tool in the database (http://pubMLST.org/chlamydiales/). Molecular Evolutionary Genetics Analysis software, version 6 (MEGA 6; http://www.megasoftware.net) was used to align all sequences based on codons in order to calculate average pairwise diversity between isolates [41]. For each gene, *p-*distance values estimated, both on the nucleotide and amino acid level, with pairwise deletion option selected and standard error (SE) determined with 1000 bootstrap replications. Using MEGA 6, average numbers of synonymous substitutions per synonymous site (dS) and non-synonymous substitutions per non-synonymous site (dN) were calculated by using the overall mean Kumar model [42, 43]. For dN/dS > 1 the Z-test of positive selection was applied and values of *P* < 0.05 were considered significant. To set a context for the Ct biological clock, dN/dS ratios were also determined for seven housekeeping genes included in the Chlamydiales MLST scheme [11] and compared these to the dN/dS ratios observed for the plasmid genes.

## Results

### Ct *core genome analyses*

Ct WGS data available in the *Chlamydiales* pubMLST database, were filtered to identify those which included complete plasmid sequence data, resulting in 157 isolates. Isolates dated from 1959 to 2011 and were from diverse geographical locations (Additional file 1: Table S1).

A total of 31 allelic *ompA* variants corresponded to 13 genovars: A, 9.6% (*n* = 15 isolates); B, 3.2% (*n* = 5); C, 1.9% (*n* = 3); D, 8.9% (*n* = 14); E, 26.8% (*n* = 42); F, 8.9% (n = 14); G, 8.3% (*n* = 13); H, 2.5% (n = 4); I, 4.5% (*n* = 7); J, 1.9% (n = 3); K, 9.6% (n = 15); L1, 3.8% (*n* = 6), and L2b, 10.2% (*n* = 16).

**Fig. 1:**
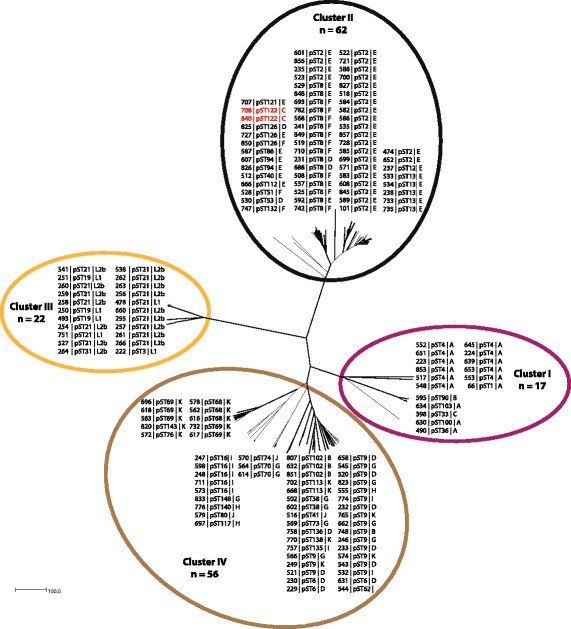
NeighborNet tree showing the core genome clustering of 157 *Chlamydia trachomatis* isolates. Coloured halos indicate the distinct clusters, isolates are indicated by the Chlamydiales pubMLST ID numbers, plasmid sequence type (pST) and *ompA* genovar. Isolates shown in red (in cluster II) indicate potential recombinants

A total of 888 out of 902 loci (98.4%) were found to be shared among 95% of the 157 isolates and represented the Ct core genome (cgMLST). Based on the diversity, a distance matrix was calculated from which a NeighborNet tree was generated (Fig. 1, Additional file 3: Table S3). Four phylogenetically distinct clusters were observed, consistent with previous studies [9]. These included: Cluster I, comprising the ocular genovars A, B and C; Cluster II, the clinically more prevalent urogenital genovars D, E and F; Cluster III, the LGV genovars L1 and L2b and, Cluster IV, the rarer urogenital genovars B, D, G, H, I, J and K. WGS from two trachoma isolates (708 and 840, both genovar C), however, clustered with urogenital Ct strains in Cluster II (Fig. 1) consistent with horizontal gene transfer. Identical clusters were identified from the maximum likelihood phylogenetic analysis (Additional file 4: Figure S1).

The number of alleles present for each core gene varied from 1 to 44 (Additional file 5: Table S4). CHAM0061 (*fliA)* encoding a Sigma-28/WhiG family protein had the lowest number of allelic variants (*n* = 1), while the highest number of alleles (*n* = 44) was observed for CHLAM0147 encoding a hypothetical protein associated with the type III secretion system (T3SS) [44]. Overall, the most diverse genes were *ompA* (CHLAM0681), the polymorphic outer membrane proteins (CHLAM0412, CHLAM0413, CHLAM0414, CHLAM0812, CHLAM0869, CHLAM0870, CHLAM0871, CHLAM0872, and CHLAM0874), 11 genes associated with the T3SS and 4 genes located in the plasticity zone (CHLAM0153, CHLAM0154, CHLAM0157 and CHLAM0166) in accordance to what was described previously [44, 45]. The plasticity zone (CHLAM0152-CHLAM0177) is a region in the Ct genome which has undergone genetic reorganization to a greater extent than the rest of the chromosome and encodes enzymes required for the biosynthesis of tryptophan [46]. The more conserved genes were found to be hypothetical proteins, RNA associated genes, genes involved in DNA replication and nucleotide excision repair.

The remaining 14 genes not included in the core genome were considered to be accessory genes. Of these, 8 comprised the plasmid genes *pgp1* to *pgp8* that were considered accessory to assess the association between the core genome and plasmid types. Although, in this dataset, all 8 plasmid genes were present, plasmids are commonly considered to be accessory since 6.5% of Ct isolates have been described to lack plasmids [47]. The remaining 6: CHLAM0165, CHLAM0166, CHLAM0167, CHLAM0173, and CHLAM0174 encoded hypothetical proteins, while CHLAM0456 encoded the translocated actin recruiting phosphoprotein (*tarp*), a T3SS effector in Chlamydia [45]. The *tarp gene* has previously been reported as highly variable correlating with ocular, urogenital and LGV disease phenotypes [44, 45, 48]. Among isolates included in this study, *tarp* alleles were present in 93/157 isolates (59.2%) with a total of 36 unique allelic variants (Additional file 6: Table S6). Analysis of *tarp* allelic variation revealed that 28/36 (77.8%) of variants were associated with disease phenotype (ocular, urogenital or LGV). For the remaining 8, alleles 25, 33 and 36 were found in WGS data from ocular, urogenital and LGV isolates, alleles 20, 22 and 44 were shared between both ocular and urogenital isolates, allele 1 was found in urogenital and LGV isolates while allele 13 was associated with an ocular and LGV isolate. Overall, the majority of *tarp* alleles were specific to isolates belonging to a particular disease phenotype, although some *tarp* alleles were also exchanged between isolates belonging to different disease phenotypes.

Analysis of *p-*distance values across the core genome revealed that the following were the most divers core genome loci: DNA-binding protein CHLAM0046 (*hctB*, *p-*distance = 0.451), ribulose-phosphate 3-epimerase CHLAM0121 (*araD*, *p-*distance = 0.500), rRNA methylase CHLAM0133 (*p-*distance = 0.063), the plasticity zone genes: CHLAM0155 (*p-*distance = 0.011), CHLAM0157 (*p-*distance = 0.099), CHLAM0171 (*p-*distance = 0.064) and hypothetical protein CHLAM0326 (*p-*distance = 0.039) (Table 1).

### Ct plasmid analyses

The gene *pgp4* (CHLAM0900) was the most conserved plasmid gene with 3 alleles containing only 2 polymorphic sites (Table 2). In contrast, *pgp6* (CHLAM902) was the most diverse, with 17 allelic variants, followed by: *pgp2* with 14, *pgp3* and *pgp1* with 12, *pgp5* and *pgp7* with 13 and *pgp8* with 11 unique allelic variants. In addition, all plasmid genes had between 15 and 23 polymorphic sites except for *pgp4*. Further analysis revealed that *pgp3* (CHLAM0899), which has also been described as a virulence-associated gene [21, 27, 28], was the most polymorphic (*p-*distance = 0.008; SE ± 0.002 and amino acid level 0.016; SE ± 0.004). Although the majority of the ORFs in the plasmid are known to be non-coding, in order to identify any putative associations with lineages or understand where such associations stemmed from, we sought to investigate whether selection pressure was evident in any of these ORFs. Therefore, the ratio between the rate of non-synonymous (dN) and synonymous (dS) substitutions per (non-) synonymous nucleotide site was determined. This ranged from 0.083 (*pgp8*) to 2.00 (*pgp5*) indicating that plasmid genes were not subject to strong positive selection. Gene *pgp5* which is suggested to regulate the expression of some chromosomal genes, showed a dN/dS = 2. We subsequently tested the null hypothesis of no selection (H0: dN  =  dS) versus the positive selection hypothesis (H1: dN > dS) using the Z-test: Z  =  (dN − dS)/√(Var(dS) + Var(dN)), but this value was not statistically significant and therefore did not indicate positive selection. The dN/dS ratios from the plasmid genes (0.083 to 2.00) were similar to those observed for theseven housekeeping genes included in the Chlamydiales MLST scheme (*gatA, oppA, hflX, gidA, enoA, hemN, and fumC*) highlighting the sequence conservation seen in the plasmid genes. Ratios from these housekeeping genes ranged from 0.167 (*fumC*) to 1.500 (*oppA_3*). Gene *oppA_3* showed a dN/dS = 1.500, but this value was not statistically significant on the Z-test and therefore did not indicate positive selection (Table 3).

**Table 1:** Genomic diversity for 888 core loci of 157 Chlamydia trachomatis isolates

Loci^a^	*p-*distance
CHLAM0046	0.451
CHLAM0121	0.500
CHLAM0133	0.063
CHALM0155	0.011
CHALM0157	0.099
CHLAM0171	0.064
CHLAM0326	0.039

^a^The remaining 881 loci had a *p-*distance< 0.01

**Table 2:** Polymorphism analysis and allelic variance of the plasmid genes derived from 157 Chlamydia trachomatis isolates

				Overall Mean Distance (pairwise deletion; bootstrap = 1000 replicates)												
				Nucleotide				Amino Acid				P-distance; Non- and Synonymous substitutions				
Gene (ORF)^a^	Length (bp)^b^	No. alleles	No. polymorphic sites	No. of differences	[SE]	P-distance	[SE]	No. of differences	[SE]	P-distance	[SE]	Mean dS	[SE]	Mean dN	[SE]	dN/dS^c^
*pgp1* (ORF3)	1356	12	18	4.965	1.402	0.004	0.001	1.602	0.682	0.004	0.002	0.007	0.002	0.002	0.001	0.286
*pgp2* (ORF4)	1065	14	20	4.420	1.125	0.004	0.001	1.043	0.553	0.003	0.002	0.009	0.003	0.001	0.001	0.111
*pgp3* (ORF5)	795	12	23	6.133	1.488	0.008	0.002	4.172	1.122	0.016	0.004	0.007	0.003	0.007	0.002	1.000
*pgp4* (ORF6)	309	3	2	0.363	0.262	0.001	0.001	0.243	0.239	0.002	0.002	0.001	0.001	0.001	0.001	1.000
*pgp5* (ORF7)	795	13	15	2.720	0.934	0.003	0.001	2.111	0.872	0.008	0.003	0.002	0.001	0.004	0.002	2.000
*pgp6* (ORF8)	744	17	20	3.003	1.020	0.004	0.001	1.415	0.648	0.006	0.003	0.006	0.003	0.003	0.001	0.500
*pgp7* (ORF1)	918	13	23	4.523	1.227	0.005	0.001	1.715	0.739	0.006	0.002	0.010	0.004	0.002	0.001	0.200
*pgp8* (ORF2)	993	11	18	4.346	1.223	0.004	0.001	0.476	0.428	0.001	0.001	0.012	0.004	0.001	0.001	0.083

^a^ORFs designation according to Thomas et al., 1997. Microbiology. 143, 1847–1854

^b^Length in base pairs relative to the sequence of the plasmid of the E/Bour strain (GenBank No. HE603212)

^c^The value of dN/dS > 1 obtained for pgp5 was not statistically significant by the Z-test of positive selection

**Table 3:** Polymorphism analysis and allelic variance among 157 *Chlamydia trachomatis* isolates of seven housekeeping genes present in the Chlamydiales MLST scheme

				Overall Mean Distance (pairwise deletion; bootstrap = 1000 replicates)												
				Nucleotide				Amino Acid				P-distance; Non- and Synonymous substitutions				
Gene^a^	Length (bp)^b^	No. alleles	No. polymorphic sites	No. of differences	[SE]	P-distance	[SE]	No. of differences	[SE]	P-distance	[SE]	Mean dS	[SE]	Mean dN	[SE]	dN/dS^c^
*enoA* (CHLAM0587)	1275	5	7	1.847	0.915	0.001	0.001	0.867	0.554	0.002	0.001	0.002	0.002	0.001	0.001	0.500
*fumC* (CHLAM0855)	1392	17	20	3.040	1.136	0.002	0.001	0.358	0.111	0.001	0.000	0.006	0.002	0.001	0.000	0.167
*gatA* (CHLAM0003)	1476	11	10	1.464	0.686	0.001	0.000	0.503	0.450	0.001	0.001	0.002	0.001	0.001	0.000	0.500
*gidA* (CHLAM0498)	1833	15	22	5.933	1.576	0.003	0.001	2.652	1.014	0.004	0.002	0.005	0.002	0.002	0.001	0.400
*hemN* (CHLAM0746)	1374	8	7	1.282	0.633	0.001	0.000	0.880	0.560	0.002	0.001	0.001	0.001	0.001	0.001	1.000
*hflX* (CHLAM0379)	1344	16	20	3.330	1.059	0.002	0.001	2.158	0.836	0.005	0.002	0.002	0.001	0.002	0.001	1.000
*oppA_3* (CHLAM0193)	1557	12	20	4.469	1.353	0.003	0.001	3.314	1.182	0.006	0.002	0.002	0.001	0.003	0.001	1.500

^a^CHLAM refers to the gene annotated in the pubMLST database

^b^Length in base pairs relative to the sequence of the plasmid of the E/Bour strain (GenBank No. HE603212)

^c^The value of dN/dS > 1 obtained for oppA_3 was not statistically significant by the Z-test of positive selection

**Fig. 2:**
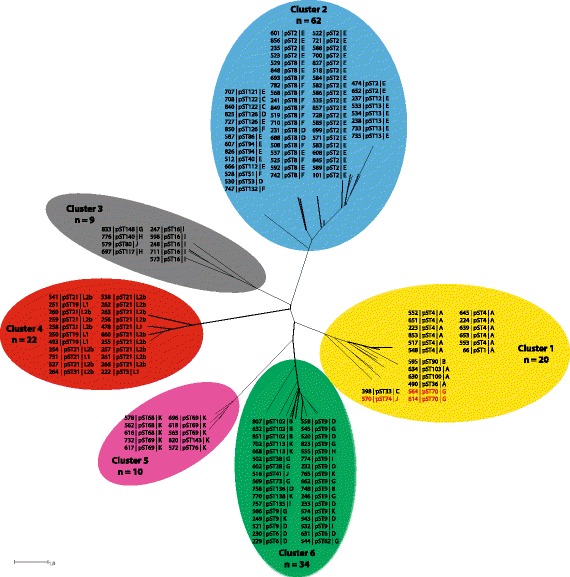
NeighborNet tree showing clustering of the plasmid sequence types (pST) generated from plasmid genes *pgp1 to pgp8* of 157 *Chlamydia trachomatis* isolates. Coloured halos indicate the distinct clusters, isolates are indicated by the Chlamydiales pubMLST ID numbers, pST and *ompA* genovar. Isolates shown in red (cluster 1) indicate potential plasmid exchange compared to the core genome clusters

**Fig. 3:**
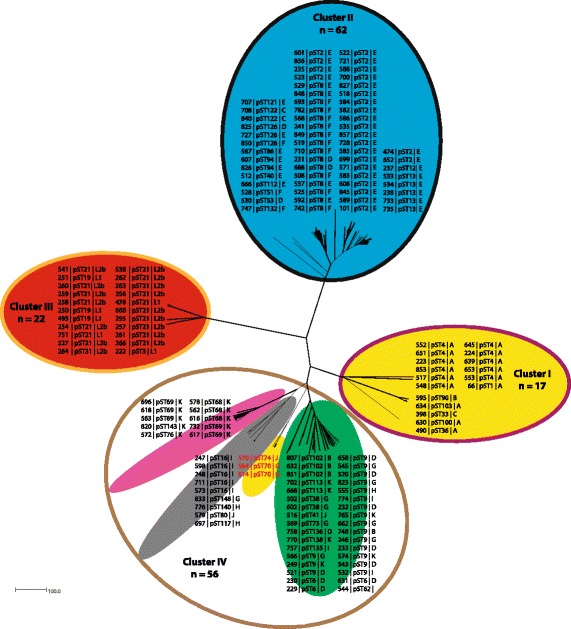
NeighborNet tree showing the comparison between core genome and plasmid clustering of 157 Chlamydia trachomatis isolates. Coloured halos indicate the distinct clusters from Fig. 1, isolates are indicated by the Chlamydiales pubMLST ID numbers, plasmid sequence type (pST) and *ompA* genovar. Isolates shown in red indicate potential plasmid exchanges compared to the plasmid clusters in Fig. 2

**Fig. 4:**
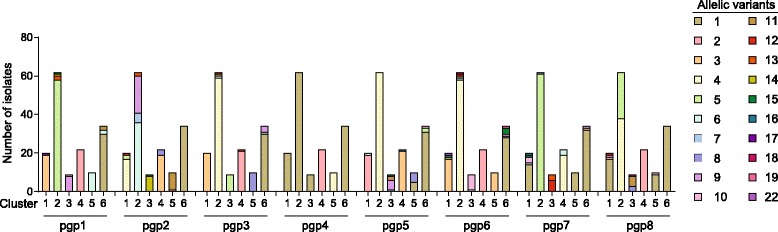
Graph showing the allelic variants for each single plasmid gene (*pgp1–8*) with respect to the observed plasmid clusters (cluster 1 to 6) among 157 *Chlamydia trachomatis* isolates. Each allelic variant received a unique colour as shown in the colour legend. Allelic numbers were randomly assigned to each unique allelic variant of a single plasmid gene (*pgp*1–8). Specific allelic numbers can be assigned to multiple plasmid genes

A total of 47 unique pSTs were randomly assigned (Additional file 3: Table S3, Fig. 1 and Additional file 4: Figure S1). Sequence comparison of plasmid genes identified six phylogenetic clusters numbered 1 to 6 (Fig. 2 and Additional file 7: Figure S2). These clusters were associated with core genome clusters; all isolates of plasmid cluster 2, 4, and 6 fell into core genome clusters II, III and IV, respectively (Fig. 3 and Additional file 8: Figure S3). All isolates except three (564, 570, and 614), of plasmid cluster 1 grouped with those from core genome cluster I. The three exceptions grouped with isolates of core genome cluster IV, suggesting horizontal plasmid transfer. In addition, plasmid clusters 3 and 6 formed sub-branches in core genome cluster IV. Overall, each core genome cluster had one dominating pST (Figs. 1 and 4, Additional file 3: Table S3). Potential recombination events were further assessed using ClonalFrameML, which results indicating that this was restricted to isolates within clusters with recombination apparent to four particular regions to the genome (Additional file 9: Figure S4).

Analysis of the allelic variation in each single plasmid gene (*pgp1* to *pgp8*) with respect to the observed plasmid clusters revealed that exchange of alleles between plasmid clusters was limited. Plasmid clusters possessed specific *pgp1, 2, 3*, and *5* alleles that were unique to each cluster (Fig. 4, Additional file 10: Table S5). For example, plasmids in cluster 1 contained *pgp1* alleles 3 and 17 which were not found in any of the other plasmid clusters, while cluster 3 had *pgp1* alleles 9 and 10. The allelic variation of *pgp6, 7* and *8* was specific for the clinically most prevalent urogenital cluster (cluster 2), one cluster containing some of the less frequently occurring genital genovars (cluster 3) and the LGV cluster (cluster 4), but exchange of allelic variants was observed among clusters 1, 5 and 6. Finally, the allelic variation of the *pgp4* gene was only specific for the LGV cluster (cluster 4) and a urogenital cluster containing the less frequently occurring genovar K isolates (cluster 5), but exchange of *pgp4* allelic variants was observed among clusters 1, 2, 3 and 6.

## Discussion

Advances in sequencing technologies and increasing availability of WGS data provide unique opportunities for improving our understanding of Ct infection and epidemiology. Pivotal to this is the ability to rapidly extract strain information from WGS data including *ompA* genovar, plasmid type and MLST sequence type such that global surveillance of Ct infections can be achieved. In this study, a catalogue of genes both core and accessory to the Ct genome was generated on the web-accessible http://pubMLST.org/chlamydiales/ website providing tools for Ct surveillance in an open database. Data presented here reveal that Ct core genomes were strongly associated with distinct Ct plasmid types (Figs. 1, 2, 4 and Additional files 4 and 7: Figures S1 and S2). Four core genome clusters were apparent following cgMLST analyses consistent with a previous study [9], however, two recombinant isolates were also apparent. These were two trachoma isolates with urogenital backbones that had been identified previously by Andersson et al. [30].

Ct is known to have a closed conserved pan-genome due to persistence of these bacteria in isolated niches with limited access to the global microbial gene pool [49]. In this dataset, a total of 888 genes were found to be core, with only 14 accessory genes consistent with the effects of genome reduction known to have occurred in Ct [50]. All of the accessory genes encoded hypothetical proteins with the exception of the plasmid genes and CHLAM0456, which encodes *tarp*. None of the hypothetical genes correlated with disease phenotype. Subsequent analysis of *tarp* allelic variants revealed that the majority of alleles (77.8%) were associated with distinct disease phenotypes (ocular, urogenital, or LGV), although some alleles were also shared between isolates from different disease phenotypes. The gene *tarp* has been suggested to contribute to tissue tropism [44, 45, 48]. However, these results indicate that it is not the sole gene driving these phenotypes. The most variable gene was CHLAM0147 encoding a T3SS effector involved in endosomal trafficking by recruiting nutrient-rich endocytic vesicles via a non-fusogenic pathway to the chlamydial inclusion [44]. Ct T3SS are activated when Ct attaches to a host cell, after which T3SS are used to deliver an arsenal of bacterial gene-encoded effector proteins into the cytosol of the host cell [44, 48, 51]. The exact molecular mechanisms of T3SS remains to be elucidated, but it is probable that these genetically diverse T3SS genes function together to favour specific tissue tropisms [44, 45].

The most conserved gene was *fliA* encoding sigma-28, for which the exact function remains to be elucidated. It has been suggested that expression of sigma-28 occurs in response to cellular stress, such as nutrient deprivation within the chlamydial inclusion [52, 53]. Overall, the most variable core genes were *ompA*, the polymorphic outer membrane proteins, genes associated with T3SS and the plasticity zone, which all have been suggested to contribute to tissue tropism and disease severity due to their high polymorphic variation [44, 45]. The more conserved genes encoded hypothetical proteins for which no known functions have been described, but have been suggested to play an important role in the complex Ct-host interactions [44, 45]. The highly-conserved nature of these genes suggests that mutations may have deleterious effects on biological fitness. Polymorphic genes included *hctB* (CHLAM0046), *araD* (CHLAM121), CHLAM133, CHLAM0155, CHLAM0157, CHLAM172 and CHLAM326. CHLAM0046 (*hctB)* encodes a DNA-binding protein thought to mediate the chromatin compaction [54]. This gene is described to vary among Ct genovars due to internal deletions from a region of the *hctB* gene encoding lysine- and alanine-rich pentameric repeats [55]. The genes CHLAM0155, CHLAM0157 and CHLAM0172 are all part of a 20.3-kb highly polymorphic genomic region encoding toxin-like genes known as the plasticity zone [45]. The chlamydia plasticity zone has also been known to vary among genovars in accordance with known phylogenetic tissue tropism (urogenital, ocular and LGV) [44, 45]. The CHLAM0326 gene encodes a hypothetical protein without a known function, but this gene has been associated with rectal tropism of Ct genovar G isolates [56]. Finally, the genes *araD* (CHLAM0121) and CHLAM0133 are known to encode a ribulose-phosphate 3-epimerase and rRNA methylase, but variation has not been linked to specific Ct strains [57].

A total of 47 unique pSTs were identified (Fig. 2, Additional file 3: Table S3) which aggregated into six distinct plasmid clusters, five of which were comparable to those of Harris et al. [9]. The additional cluster consisted solely of genovar K isolates and was likely the result of Ct strains that had previously not been sampled. In comparison to the clusters observed following cgMLST analyses, plasmid analyses showed that the rarer *ompA* genovars (genovars B, D, G, H, I, J, and K) formed three distinct plasmid clusters. Moreover, three urogenital isolates with genovars G and J were identified that contained plasmids clustering with ocular isolates (genovars A-C), suggesting that exchange of plasmids may have occurred between urogenital and trachoma isolates. Overall, our findings are in agreement with previous studies that suggested co-evolution of Ct plasmids and their chromosome and demonstrated that, although rare, exchange of a plasmid can occur [9, 12]. In addition, allelic variation in some of the plasmid genes was specific and distinct for each cluster, while variation in other genes was mainly specific for the LGV cluster since the allelic variants of the remaining clusters were shared. Potential recombination events were further assessed using ClonalFrameML, which revealed evidence of recombination within the clusters that was restricted to four particular regions in the genome. Further examination should reveal the exact genomic location of these recombination hotspots and their subsequent contribution to *C. trachomatis* pathogenesis and evolution (Additional file 9: Figure S4).

The observed polymorphic variation was in agreement with previous analyses [9, 13, 58] and comparison to the polymorphic variation found in seven housekeeping genes included in the Chlamydiales MLST scheme highlighted the sequence conservation of the Ct plasmid genes. Although the plasmid genes were highly conserved, much of the diversity appeared to be restricted to one plasmid gene known to be associated with chlamydial virulence. This gene, *pgp3*, which is associated with increased Ct inflammatory responses [21, 27, 28], was the most polymorphic gene (*p-*distance = 0.008) and is known to be secreted in the host cell cytosol and its diversity is possibly a result of immune selection [12, 21, 27, 28]. In contrast, *pgp4,* which functions as a transcriptional regulator of *pgp3* and some chromosomally encoded genes, was identified as the most conserved gene. The high sequence conservation of this gene suggests that *pgp4* is essential for virulence and infection, although previous studies have demonstrated that *pgp4* was dispensable for growth in vitro [25, 59]. Finally, we observed that exchange of alleles between plasmid clusters was very limited. Some alleles were specific to each plasmid cluster, with, in particular, distinct plasmid alleles among LGV isolates. As Ct harbours multiple plasmid copies, these results may be useful to design plasmid assays which can distinguish clinically relevant Ct strain types, for instance to detect the Swedish truncated plasmid variant or to differentiate between LGV/non-LGV isolates. The latter is of clinical importance since LGV infections are more invasive and require extended treatment [9, 12, 60].

A limitation of our study was that it used previously sequenced and stored isolates, for which limited epidemiological or geographical data were available. Since these isolates were originally selected for different objectives, the data available could not be extrapolated to all included isolates. Previous molecular epidemiological studies on Ct using MLST showed no association between Ct strain types and symptoms, anatomical locations, gender or geographical location. Most strain types were also globally distributed with similar *ompA* genovar distributions [10, 61–63]. These studies therefore suggest that the population structure of Ct is comparable among different human populations and that the effect of selection bias would be minimal. However, MLST is limited by the fact that only a small (but polymorphic) fragment of the genome is used for typing, so samples, that are indistinguishable by MLST type, may still contain (considerable) genomic diversity in the rest of the genome. Future studies should therefore include a well-defined population with known epidemiological and clinical data to gain a better understanding of Ct epidemiology in relation to the population structure while also analysing the whol genome in association with MLST.

In conclusion, a strong association between Ct core genome and plasmid types was observed, consistent with co-evolution of Ct plasmids and their chromosome. Moreover, we suggest that, although rare, plasmid exchange may occur between isolates. Finally, we showed that exchange of alleles between plasmid clusters was limited. Future research should apply the gene-by-gene approach to a well-defined population with known epidemiological and clinical data, as this will enhance our understanding of chlamydial transmission and disease.

## Additional files

Additional file 1: Table S1. List of samples used in this study and associated metadata. (XLSX 20 kb)

Additional file 2: Table S2. List of annotated loci present in the Chlamydiales pubMLST database. (XLSX 60 kb)

Additional file 3: Table S3. Plasmid data of the 157 *C. trachomatis isolates*. Coding is according to the Chlamydiales pubMLST database (http://pubMLST.org/chlamydiales/). The samples are sorted by cluster and plasmid sequence type. (DOCX 30 kb)

Additional file 4: Figure S1. Maximum Likelihood phylogenetic tree derived from core genes of the 157 *Chlamydia trachomatis* isolates in this study (plasmid omitted) (DOCX 380 kb)

Additional file 5: Table S4. List of genome genes and functions with respect to the allelic variance. (XLSX 58 kb)

Additional file 6: Table S6. The number of allelic variants for the CHLAM0456 (*tarp*) gene in respect to disease phenotype (ocular, urogenital, LGV). The samples are sorted by allelic variant. (DOCX 15 kb)

Additional file 7: Figure S2. Maximum Likelihood phylogenetic tree derived from concatenated, aligned nucleotide sequence data from plasmid loci. (DOCX 325 kb)

Additional file 8: Figure S3. Maximum Likelihood phylogenetic tree derived from concatenated, aligned nucleotide sequence data from both core and plasmid loci. (DOCX 393 kb)

Additional file 9: Figure S4. ClonalFrameML Recombination-corrected. Maximum Likelihood phylogenetic tree derived from concatenated, aligned nucleotide sequence. Dark blue horizontal bars indicate recombination events, light blue indicates lack of substitution, and colours ranging from white to red indicate substitutions with increasing levels of homoplasy. (DOCX 857 kb)

Additional file 10: Table S5. The number of allelic variants for each plasmid gene (pgp1 to 8) in respect to the observed plasmid clusters among 157 *C. trachomatis* isolates. The samples are sorted by plasmid gene and allelic variant. (DOCX 21 kb)

## Abbreviations

BIGSdb: Bacterial Isolate Genome Sequence database
cgMLST: Core Genome MLST
Ct: *C. trachomatis*
ENA: European Nucleotide Archive
LGV: Lymphogranyloma venereum
MLST: Multilocus sequence typing
ORF: Open reading frame
pST: Plasmid Sequence Type
T3SS: Type III secretion system
*tarp*: translocated actin recruiting phosphoprotein
WGS: Whole Genome Sequences
