# Supplementary material for: Genomic analyses of the Chlamydia trachomatis core genome show an association between chromosomal genome, plasmid type and disease
Source: BMC Genomics. 2018 Feb 9;19:130. doi: 10.1186/s12864-018-4522-3 (PMC5810182; doi:10.1186/s12864-018-4522-3)
Supplement: Supplementary file 3 — Plasmid data of the 157 C. trachomatis isolates. Coding is according to the Chlamydiales pubMLST database (http://pubMLST.org/chlamydiales/). The samples are sorted by cluster and plasmid sequence type. (DOCX 30 kb) [file 12864_2018_4522_MOESM3_ESM.docx]

**Supplementary Table 3:** Plasmid data of the 157 *C. trachomatis* isolates. Coding is according to the Chlamydiales pubMLST database (<http://pubMLST.org/chlamydiales/>). The samples are sorted by cluster and plasmid sequence type.

| **Cluster name** | **ID^a^** | **pST** | ***ompA* genovar** | **pgp1** | **pgp2** | **pgp3** | **pgp4** | **pgp5** | **pgp6** | **pgp7** | **pgp8** |
| --- | --- | --- | --- | --- | --- | --- | --- | --- | --- | --- | --- |
| Cluster 1 | 66 | 1 | A | 1 | 3 | 3 | 4 | 3 | 1 | 2 | 3 |
|  | 223 | 4 | A | 1 | 1 | 3 | 4 | 3 | 1 | 2 | 3 |
|  | 224 | 4 | A | 1 | 1 | 3 | 4 | 3 | 1 | 2 | 3 |
|  | 517 | 4 | A | 1 | 1 | 3 | 4 | 3 | 1 | 2 | 3 |
|  | 548 | 4 | A | 1 | 1 | 3 | 4 | 3 | 1 | 2 | 3 |
|  | 552 | 4 | A | 1 | 1 | 3 | 4 | 3 | 1 | 2 | 3 |
|  | 553 | 4 | A | 1 | 1 | 3 | 4 | 3 | 1 | 2 | 3 |
|  | 639 | 4 | A | 1 | 1 | 3 | 4 | 3 | 1 | 2 | 3 |
|  | 645 | 4 | A | 1 | 1 | 3 | 4 | 3 | 1 | 2 | 3 |
|  | 651 | 4 | A | 1 | 1 | 3 | 4 | 3 | 1 | 2 | 3 |
|  | 653 | 4 | A | 1 | 1 | 3 | 4 | 3 | 1 | 2 | 3 |
|  | 853 | 4 | A | 1 | 1 | 3 | 4 | 3 | 1 | 2 | 3 |
|  | 398 | 33 | C | 7 | 1 | 17 | 4 | 3 | 1 | 2 | 22 |
|  | 490 | 36 | A | 1 | 1 | 3 | 4 | 3 | 1 | 6 | 3 |
|  | 564 | 70 | G | 10 | 1 | 3 | 5 | 3 | 1 | 2 | 3 |
|  | 614 | 70 | G | 10 | 1 | 3 | 5 | 3 | 1 | 2 | 3 |
|  | 570 | 74 | J | 10 | 9 | 3 | 12 | 3 | 1 | 2 | 3 |
|  | 595 | 90 | B | 15 | 12 | 3 | 4 | 3 | 1 | 2 | 3 |
|  | 630 | 100 | A | 1 | 1 | 3 | 4 | 3 | 1 | 2 | 14 |
|  | 634 | 103 | A | 16 | 1 | 3 | 4 | 3 | 1 | 2 | 16 |
| Cluster 2 | 101 | 2 | E | 5 | 4 | 5 | 6 | 4 | 1 | 4 | 4 |
|  | 235 | 2 | E | 5 | 4 | 5 | 6 | 4 | 1 | 4 | 4 |
|  | 474 | 2 | E | 5 | 4 | 5 | 6 | 4 | 1 | 4 | 4 |
|  | 518 | 2 | E | 5 | 4 | 5 | 6 | 4 | 1 | 4 | 4 |
|  | 522 | 2 | E | 5 | 4 | 5 | 6 | 4 | 1 | 4 | 4 |
|  | 523 | 2 | E | 5 | 4 | 5 | 6 | 4 | 1 | 4 | 4 |
|  | 535 | 2 | E | 5 | 4 | 5 | 6 | 4 | 1 | 4 | 4 |
|  | 571 | 2 | E | 5 | 4 | 5 | 6 | 4 | 1 | 4 | 4 |
|  | 582 | 2 | E | 5 | 4 | 5 | 6 | 4 | 1 | 4 | 4 |
|  | 583 | 2 | E | 5 | 4 | 5 | 6 | 4 | 1 | 4 | 4 |
|  | 584 | 2 | E | 5 | 4 | 5 | 6 | 4 | 1 | 4 | 4 |
|  | 585 | 2 | E | 5 | 4 | 5 | 6 | 4 | 1 | 4 | 4 |
|  | 586 | 2 | E | 5 | 4 | 5 | 6 | 4 | 1 | 4 | 4 |
|  | 588 | 2 | E | 5 | 4 | 5 | 6 | 4 | 1 | 4 | 4 |
|  | 589 | 2 | E | 5 | 4 | 5 | 6 | 4 | 1 | 4 | 4 |
|  | 601 | 2 | E | 5 | 4 | 5 | 6 | 4 | 1 | 4 | 4 |
|  | 608 | 2 | E | 5 | 4 | 5 | 6 | 4 | 1 | 4 | 4 |
|  | 652 | 2 | E | 5 | 4 | 5 | 6 | 4 | 1 | 4 | 4 |
|  | 699 | 2 | E | 5 | 4 | 5 | 6 | 4 | 1 | 4 | 4 |
|  | 700 | 2 | E | 5 | 4 | 5 | 6 | 4 | 1 | 4 | 4 |
|  | 721 | 2 | E | 5 | 4 | 5 | 6 | 4 | 1 | 4 | 4 |
|  | 728 | 2 | E | 5 | 4 | 5 | 6 | 4 | 1 | 4 | 4 |
|  | 827 | 2 | E | 5 | 4 | 5 | 6 | 4 | 1 | 4 | 4 |
|  | 845 | 2 | E | 5 | 4 | 5 | 6 | 4 | 1 | 4 | 4 |
|  | 856 | 2 | E | 5 | 4 | 5 | 6 | 4 | 1 | 4 | 4 |
|  | 857 | 2 | E | 5 | 4 | 5 | 6 | 4 | 1 | 4 | 4 |
|  | 231 | 8 | D | 5 | 5 | 5 | 9 | 4 | 1 | 4 | 4 |
|  | 241 | 8 | F | 5 | 5 | 5 | 9 | 4 | 1 | 4 | 4 |
|  | 508 | 8 | F | 5 | 5 | 5 | 9 | 4 | 1 | 4 | 4 |
|  | 519 | 8 | F | 5 | 5 | 5 | 9 | 4 | 1 | 4 | 4 |
|  | 525 | 8 | F | 5 | 5 | 5 | 9 | 4 | 1 | 4 | 4 |
|  | 529 | 8 | F | 5 | 5 | 5 | 9 | 4 | 1 | 4 | 4 |
|  | 537 | 8 | E | 5 | 5 | 5 | 9 | 4 | 1 | 4 | 4 |
|  | 568 | 8 | F | 5 | 5 | 5 | 9 | 4 | 1 | 4 | 4 |
|  | 592 | 8 | E | 5 | 5 | 5 | 9 | 4 | 1 | 4 | 4 |
|  | 688 | 8 | D | 5 | 5 | 5 | 9 | 4 | 1 | 4 | 4 |
|  | 693 | 8 | F | 5 | 5 | 5 | 9 | 4 | 1 | 4 | 4 |
|  | 710 | 8 | F | 5 | 5 | 5 | 9 | 4 | 1 | 4 | 4 |
|  | 742 | 8 | F | 5 | 5 | 5 | 9 | 4 | 1 | 4 | 4 |
|  | 782 | 8 | F | 5 | 5 | 5 | 9 | 4 | 1 | 4 | 4 |
|  | 848 | 8 | E | 5 | 5 | 5 | 9 | 4 | 1 | 4 | 4 |
|  | 849 | 8 | F | 5 | 5 | 5 | 9 | 4 | 1 | 4 | 4 |
|  | 237 | 12 | E | 5 | 4 | 5 | 6 | 4 | 1 | 4 | 11 |
|  | 238 | 13 | E | 5 | 4 | 5 | 7 | 4 | 1 | 4 | 4 |
|  | 533 | 13 | E | 5 | 4 | 5 | 7 | 4 | 1 | 4 | 4 |
|  | 534 | 13 | E | 5 | 4 | 5 | 7 | 4 | 1 | 4 | 4 |
|  | 733 | 13 | E | 5 | 4 | 5 | 7 | 4 | 1 | 4 | 4 |
|  | 735 | 13 | E | 5 | 4 | 5 | 7 | 4 | 1 | 4 | 4 |
|  | 512 | 40 | E | 5 | 4 | 5 | 6 | 6 | 1 | 4 | 4 |
|  | 528 | 51 | F | 8 | 5 | 5 | 9 | 4 | 1 | 4 | 4 |
|  | 530 | 53 | D | 5 | 5 | 5 | 9 | 4 | 1 | 4 | 7 |
|  | 587 | 86 | E | 5 | 4 | 5 | 6 | 4 | 1 | 4 | 12 |
|  | 607 | 94 | E | 5 | 4 | 5 | 13 | 4 | 1 | 4 | 4 |
|  | 826 | 94 | E | 5 | 4 | 5 | 13 | 4 | 1 | 4 | 4 |
|  | 666 | 112 | E | 5 | 4 | 14 | 6 | 4 | 1 | 4 | 4 |
|  | 707 | 121 | E | 5 | 4 | 5 | 6 | 11 | 1 | 4 | 18 |
|  | 708 | 122 | C | 5 | 5 | 13 | 6 | 4 | 1 | 4 | 4 |
|  | 840 | 122 | C | 5 | 5 | 13 | 6 | 4 | 1 | 4 | 4 |
|  | 727 | 126 | E | 5 | 5 | 5 | 6 | 4 | 1 | 4 | 4 |
|  | 825 | 126 | D | 5 | 5 | 5 | 6 | 4 | 1 | 4 | 4 |
|  | 850 | 126 | F | 5 | 5 | 5 | 6 | 4 | 1 | 4 | 4 |
|  | 747 | 132 | F | 5 | 5 | 15 | 9 | 12 | 1 | 4 | 4 |
| Cluster 3 | 247 | 16 | I | 12 | 11 | 9 | 14 | 5 | 1 | 9 | 10 |
|  | 248 | 16 | I | 12 | 11 | 9 | 14 | 5 | 1 | 9 | 10 |
|  | 573 | 16 | I | 12 | 11 | 9 | 14 | 5 | 1 | 9 | 10 |
|  | 598 | 16 | I | 12 | 11 | 9 | 14 | 5 | 1 | 9 | 10 |
|  | 711 | 16 | I | 12 | 11 | 9 | 14 | 5 | 1 | 9 | 10 |
|  | 579 | 80 | J | 13 | 8 | 9 | 14 | 5 | 1 | 7 | 10 |
|  | 697 | 117 | H | 13 | 8 | 9 | 14 | 5 | 1 | 11 | 10 |
|  | 776 | 140 | H | 13 | 8 | 10 | 14 | 5 | 1 | 11 | 10 |
|  | 833 | 148 | G | 12 | 18 | 9 | 15 | 5 | 1 | 15 | 6 |
| Cluster 4 | 222 | 3 | L1 | 4 | 2 | 2 | 3 | 2 | 2 | 16 | 2 |
|  | 250 | 19 | L1 | 6 | 2 | 2 | 8 | 2 | 2 | 3 | 2 |
|  | 251 | 19 | L1 | 6 | 2 | 2 | 8 | 2 | 2 | 3 | 2 |
|  | 493 | 19 | L1 | 6 | 2 | 2 | 8 | 2 | 2 | 3 | 2 |
|  | 254 | 21 | L2b | 4 | 2 | 2 | 3 | 2 | 2 | 3 | 2 |
|  | 255 | 21 | L2b | 4 | 2 | 2 | 3 | 2 | 2 | 3 | 2 |
|  | 256 | 21 | L2b | 4 | 2 | 2 | 3 | 2 | 2 | 3 | 2 |
|  | 257 | 21 | L2b | 4 | 2 | 2 | 3 | 2 | 2 | 3 | 2 |
|  | 258 | 21 | L2b | 4 | 2 | 2 | 3 | 2 | 2 | 3 | 2 |
|  | 259 | 21 | L2b | 4 | 2 | 2 | 3 | 2 | 2 | 3 | 2 |
|  | 260 | 21 | L2b | 4 | 2 | 2 | 3 | 2 | 2 | 3 | 2 |
|  | 261 | 21 | L2b | 4 | 2 | 2 | 3 | 2 | 2 | 3 | 2 |
|  | 262 | 21 | L2b | 4 | 2 | 2 | 3 | 2 | 2 | 3 | 2 |
|  | 263 | 21 | L2b | 4 | 2 | 2 | 3 | 2 | 2 | 3 | 2 |
|  | 266 | 21 | L2b | 4 | 2 | 2 | 3 | 2 | 2 | 3 | 2 |
|  | 478 | 21 | L1 | 4 | 2 | 2 | 3 | 2 | 2 | 3 | 2 |
|  | 527 | 21 | L2b | 4 | 2 | 2 | 3 | 2 | 2 | 3 | 2 |
|  | 538 | 21 | L2b | 4 | 2 | 2 | 3 | 2 | 2 | 3 | 2 |
|  | 541 | 21 | L2b | 4 | 2 | 2 | 3 | 2 | 2 | 3 | 2 |
|  | 660 | 21 | L2b | 4 | 2 | 2 | 3 | 2 | 2 | 3 | 2 |
|  | 751 | 21 | L1 | 4 | 2 | 2 | 3 | 2 | 2 | 3 | 2 |
|  | 264 | 31 | L2b | 4 | 2 | 2 | 3 | 13 | 2 | 3 | 2 |
| Cluster 5 | 562 | 68 | K | 1 | 1 | 6 | 11 | 8 | 4 | 1 | 3 |
|  | 578 | 68 | K | 1 | 1 | 6 | 11 | 8 | 4 | 1 | 3 |
|  | 616 | 68 | K | 1 | 1 | 6 | 11 | 8 | 4 | 1 | 3 |
|  | 563 | 69 | K | 1 | 1 | 6 | 11 | 8 | 4 | 8 | 3 |
|  | 617 | 69 | K | 1 | 1 | 6 | 11 | 8 | 4 | 8 | 3 |
|  | 618 | 69 | K | 1 | 1 | 6 | 11 | 8 | 4 | 8 | 3 |
|  | 696 | 69 | K | 1 | 1 | 6 | 11 | 8 | 4 | 8 | 3 |
|  | 732 | 69 | K | 1 | 1 | 6 | 11 | 8 | 4 | 8 | 3 |
|  | 572 | 76 | K | 1 | 10 | 6 | 11 | 8 | 4 | 1 | 3 |
|  | 820 | 143 | K | 1 | 1 | 6 | 2 | 8 | 4 | 1 | 3 |
| Cluster 6 | 229 | 6 | D | 1 | 1 | 1 | 1 | 9 | 1 | 1 | 1 |
|  | 230 | 6 | D | 1 | 1 | 1 | 1 | 9 | 1 | 1 | 1 |
|  | 631 | 6 | D | 1 | 1 | 1 | 1 | 9 | 1 | 1 | 1 |
|  | 232 | 9 | D | 1 | 1 | 1 | 1 | 1 | 1 | 1 | 1 |
|  | 233 | 9 | D | 1 | 1 | 1 | 1 | 1 | 1 | 1 | 1 |
|  | 246 | 9 | G | 1 | 1 | 1 | 1 | 1 | 1 | 1 | 1 |
|  | 249 | 9 | K | 1 | 1 | 1 | 1 | 1 | 1 | 1 | 1 |
|  | 520 | 9 | D | 1 | 1 | 1 | 1 | 1 | 1 | 1 | 1 |
|  | 521 | 9 | D | 1 | 1 | 1 | 1 | 1 | 1 | 1 | 1 |
|  | 532 | 9 | I | 1 | 1 | 1 | 1 | 1 | 1 | 1 | 1 |
|  | 543 | 9 | D | 1 | 1 | 1 | 1 | 1 | 1 | 1 | 1 |
|  | 545 | 9 | G | 1 | 1 | 1 | 1 | 1 | 1 | 1 | 1 |
|  | 555 | 9 | H | 1 | 1 | 1 | 1 | 1 | 1 | 1 | 1 |
|  | 566 | 9 | G | 1 | 1 | 1 | 1 | 1 | 1 | 1 | 1 |
|  | 574 | 9 | K | 1 | 1 | 1 | 1 | 1 | 1 | 1 | 1 |
|  | 658 | 9 | D | 1 | 1 | 1 | 1 | 1 | 1 | 1 | 1 |
|  | 662 | 9 | G | 1 | 1 | 1 | 1 | 1 | 1 | 1 | 1 |
|  | 748 | 9 | B | 1 | 1 | 1 | 1 | 1 | 1 | 1 | 1 |
|  | 765 | 9 | K | 1 | 1 | 1 | 1 | 1 | 1 | 1 | 1 |
|  | 774 | 9 | H | 1 | 1 | 1 | 1 | 1 | 1 | 1 | 1 |
|  | 823 | 9 | G | 1 | 1 | 1 | 1 | 1 | 1 | 1 | 1 |
|  | 502 | 38 | G | 1 | 1 | 6 | 1 | 1 | 1 | 5 | 1 |
|  | 602 | 38 | G | 1 | 1 | 6 | 1 | 1 | 1 | 5 | 1 |
|  | 516 | 41 | J | 1 | 1 | 1 | 1 | 7 | 1 | 1 | 1 |
|  | 544 | 62 | G | 9 | 1 | 1 | 1 | 1 | 1 | 1 | 8 |
|  | 569 | 73 | G | 1 | 1 | 1 | 1 | 1 | 1 | 1 | 9 |
|  | 632 | 102 | B | 1 | 1 | 1 | 1 | 1 | 1 | 1 | 15 |
|  | 807 | 102 | B | 1 | 1 | 1 | 1 | 1 | 1 | 1 | 15 |
|  | 851 | 102 | B | 1 | 1 | 1 | 1 | 1 | 1 | 1 | 15 |
|  | 668 | 113 | K | 1 | 1 | 11 | 1 | 1 | 1 | 1 | 1 |
|  | 702 | 113 | G | 1 | 1 | 11 | 1 | 1 | 1 | 1 | 1 |
|  | 757 | 135 | I | 1 | 1 | 1 | 1 | 1 | 1 | 1 | 19 |
|  | 758 | 136 | D | 2 | 1 | 1 | 1 | 1 | 1 | 1 | 1 |
|  | 770 | 138 | K | 1 | 1 | 1 | 1 | 1 | 1 | 10 | 1 |
| pST: plasmid sequence type | | | | | | | | | | | |
| ^a^ All ID numbers correspond with those present in the Chlamydiales pubMLST database (<http://pubmlst.org/chlamydiales/>) | | | | | | | | | | | |
