# Supplementary material for: Genomic analyses of the Chlamydia trachomatis core genome show an association between chromosomal genome, plasmid type and disease
Source: BMC Genomics. 2018 Feb 9;19:130. doi: 10.1186/s12864-018-4522-3 (PMC5810182; doi:10.1186/s12864-018-4522-3)

**Supplementary Figure 1**. Maximum Likelihood phylogenetic tree derived from core genes of the 157 *Chlamydia trachomatis* isolates in this study (plasmid omitted)


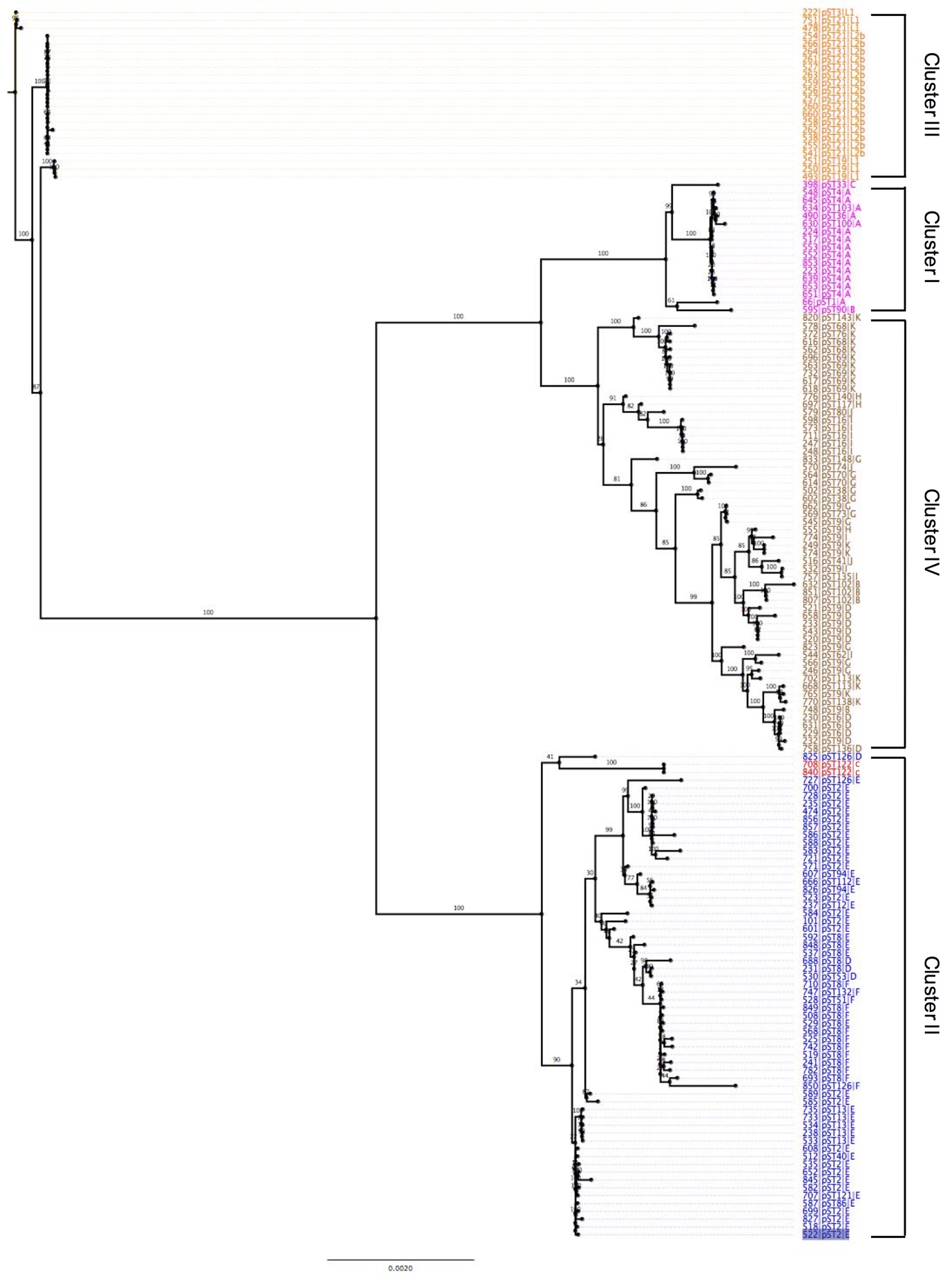

Supplement: Supplementary file 4 — Maximum Likelihood phylogenetic tree derived from core genes of the 157 Chlamydia trachomatis isolates in this study (plasmid omitted) (DOCX 380 kb) [file 12864_2018_4522_MOESM4_ESM.docx]
