# Supplementary material for: Genomic analyses of the Chlamydia trachomatis core genome show an association between chromosomal genome, plasmid type and disease
Source: BMC Genomics. 2018 Feb 9;19:130. doi: 10.1186/s12864-018-4522-3 (PMC5810182; doi:10.1186/s12864-018-4522-3)
Supplement: Supplementary file 6 — The number of allelic variants for the CHLAM0456 (tarp) gene in respect to disease phenotype (ocular, urogenital, LGV). The samples are sorted by allelic variant. (DOCX 15 kb) [file 12864_2018_4522_MOESM6_ESM.docx]

**Supplementary Table 6:** The number of allelic variants for the CHLAM0456 (*tarp*) gene in respect to disease phenotype (ocular, urogenital, LGV). The samples are sorted by allelic variant.

|  |  | **Disease phenotype** | | |
| --- | --- | --- | --- | --- |
| **Gene** | **Allelic variation** | **Ocular** | **Urogenital** | **LGV** |
| Tarp | 1 | - | 7 | 1 |
|  | 6 | - | 1 | - |
|  | 7 | - | 2 | - |
|  | 13 | 1 | - | 7 |
|  | 14 | - | - | 1 |
|  | 15 | - | - | 1 |
|  | 16 | - | - | 1 |
|  | 18 | - | 1 | - |
|  | 19 | 1 | - | - |
|  | 20 | 3 | 3 | - |
|  | 21 | - | 1 | - |
|  | 22 | 1 | 1 | - |
|  | 23 | - | 1 | - |
|  | 24 | - | 3 | - |
|  | 25 | 2 | 18 | 1 |
|  | 26 | - | 1 | - |
|  | 27 | - | - | 1 |
|  | 28 | - | 4 | - |
|  | 29 | - | 1 | - |
|  | 30 | - | - | 1 |
|  | 31 | - | - | 1 |
|  | 32 | - | 1 | - |
|  | 33 | 3 | 2 | 1 |
|  | 34 | - | 1 | - |
|  | 35 | - | 1 | - |
|  | 36 | 1 | 2 | 1 |
|  | 37 | - | 1 | - |
|  | 38 | - | 1 | - |
|  | 39 | - | 1 | - |
|  | 40 | - | 2 | - |
|  | 41 | - | 1 | - |
|  | 42 | - | 2 | - |
|  | 44 | 1 | 1 | - |
|  | 46 | - | 1 | - |
|  | 47 | - | 1 | - |
|  | 48 | 1 | - | - |
