# Supplementary material for: Genomic analyses of the Chlamydia trachomatis core genome show an association between chromosomal genome, plasmid type and disease
Source: BMC Genomics. 2018 Feb 9;19:130. doi: 10.1186/s12864-018-4522-3 (PMC5810182; doi:10.1186/s12864-018-4522-3)

**Supplementary Figure 3.** Maximum Likelihood phylogenetic tree derived from concatenated, aligned nucleotide sequence data from both core and plasmid loci.


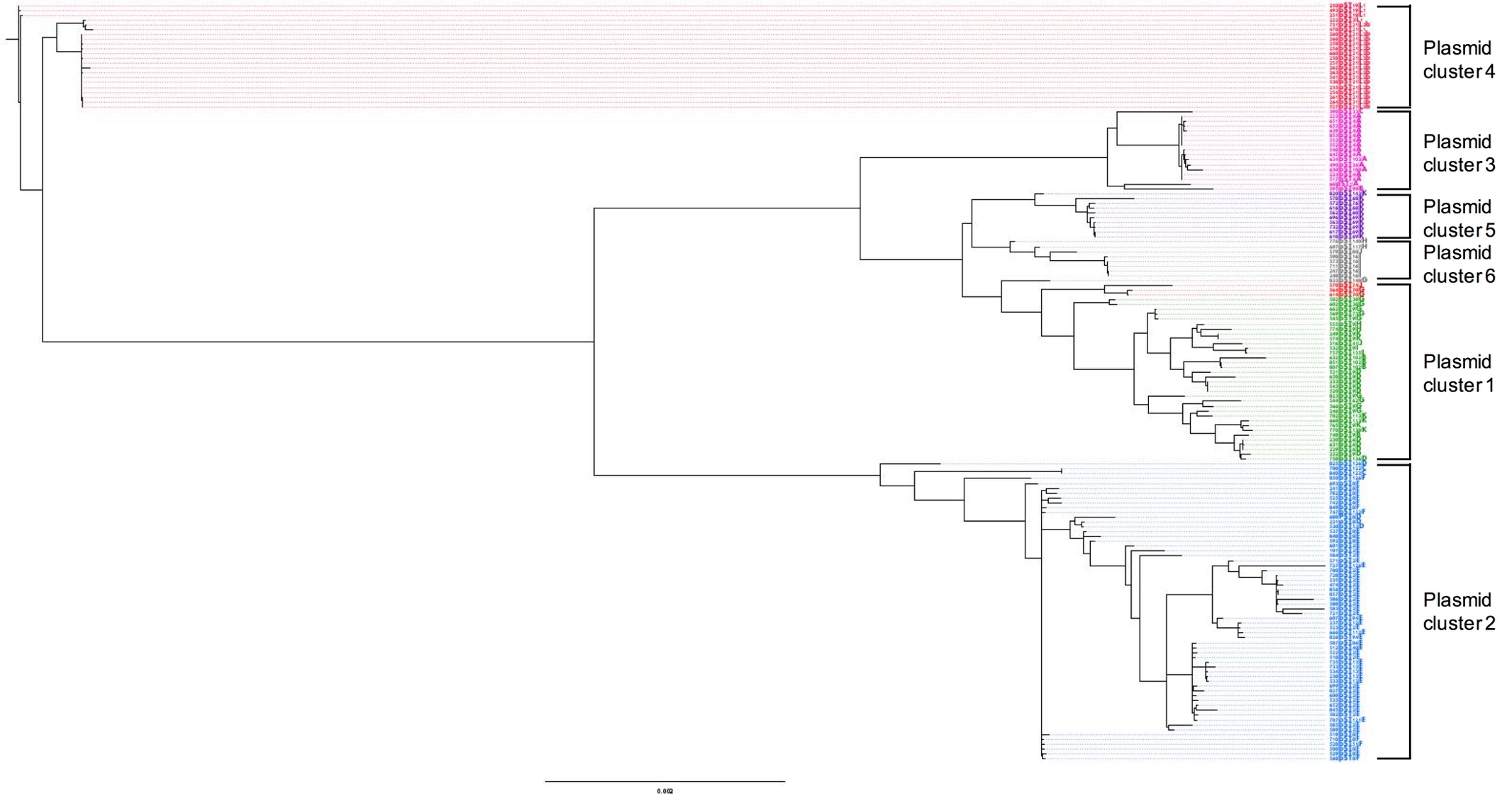

Supplement: Supplementary file 8 — Maximum Likelihood phylogenetic tree derived from concatenated, aligned nucleotide sequence data from both core and plasmid loci. (DOCX 393 kb) [file 12864_2018_4522_MOESM8_ESM.docx]
