# Supplementary material for: Genomic analyses of the Chlamydia trachomatis core genome show an association between chromosomal genome, plasmid type and disease
Source: BMC Genomics. 2018 Feb 9;19:130. doi: 10.1186/s12864-018-4522-3 (PMC5810182; doi:10.1186/s12864-018-4522-3)
Supplement: Supplementary file 9 — ClonalFrameML Recombination-corrected. Maximum Likelihood phylogenetic tree derived from concatenated, aligned nucleotide sequence. Dark blue horizontal bars indicate recombination events, light blue indicates lack of substitution, and colours ranging from white to red indicate substitutions with increasing levels of homoplasy. (DOCX 857 kb) [file 12864_2018_4522_MOESM9_ESM.docx]

**Supplementary Figure 4.** ClonalFrameML Recombination-corrected. Maximum Likelihood phylogenetic tree derived from concatenated, aligned nucleotide sequence. Dark blue horizontal bars indicate recombination events, light blue indicates lack of substitution, and colours ranging from white to red indicate substitutions with increasing levels of homoplasy.
