# Supplementary material for: Genomic analyses of the Chlamydia trachomatis core genome show an association between chromosomal genome, plasmid type and disease
Source: BMC Genomics. 2018 Feb 9;19:130. doi: 10.1186/s12864-018-4522-3 (PMC5810182; doi:10.1186/s12864-018-4522-3)
Supplement: Supplementary file 10 — The number of allelic variants for each plasmid gene (pgp1 to 8) in respect to the observed plasmid clusters among 157 C. trachomatis isolates. The samples are sorted by plasmid gene and allelic variant. (DOCX 21 kb) [file 12864_2018_4522_MOESM10_ESM.docx]

**Supplementary Table 5:** The number of allelic variants for each plasmid gene (pgp1 to 8) in respect to the observed plasmid clusters among 157 *C. trachomatis* isolates. The samples are sorted by plasmid gene and allelic variant.

| **Gene** | **Allelic variant** | **Cluster 1** | **Cluster 2** | **Cluster 3** | **Cluster 4** | **Cluster 5** | **Cluster 6** |
| --- | --- | --- | --- | --- | --- | --- | --- |
| pgp1 | 1 | - | - | - | - | - | 32 |
|  | 2 | - | - | - | 22 | - | - |
|  | 3 | 19 | - | - | - | - | - |
|  | 5 | - | 58 | - | - | - | - |
|  | 6 | - | - | - | - | 10 | - |
|  | 9 | - | - | 8 | - | - | - |
|  | 10 | - | - | 1 | - | - | - |
|  | 11 | - | - | - | - | - | 2 |
|  | 13 | - | 2 | - | - | - | - |
|  | 14 | - | 1 | - | - | - | - |
|  | 15 | - | 1 | - | - | - | - |
|  | 17 | 1 | - | - | - | - | - |
| pgp2 | 1 | - | - | - | - | - | 34 |
|  | 2 | - | - | - | - | 1 | - |
|  | 3 | - | - | - | 19 | - | - |
|  | 4 | 17 | - | - | - | - | - |
|  | 5 | 2 | - | - | - | - | - |
|  | 6 | - | 36 | - | - | - | - |
|  | 7 | - | 5 | - | - | - | - |
|  | 8 | - | - | - | 3 | - | - |
|  | 9 | - | 19 | - | - | - | - |
|  | 11 | - | - | - | - | 9 | - |
|  | 12 | 1 | - | - | - | - | - |
|  | 13 | - | 2 | - | - | - | - |
|  | 14 | - | - | 8 | - | - | - |
|  | 15 | - | - | 1 | - | - | - |
| pgp3 | 1 | - | - | - | - | - | 30 |
|  | 2 | - | - | - | 21 | - | - |
|  | 3 | 20 | - | - | - | - | - |
|  | 4 | - | 59 | - | - | - | - |
|  | 5 | - | - | 9 | - | - | - |
|  | 6 | - | 1 | - | - | - | - |
|  | 7 | - | - | - | - | - | 1 |
|  | 8 | - | - | - | - | 10 | - |
|  | 9 | - | - | - | - | - | 3 |
|  | 11 | - | 1 | - | - | - | - |
|  | 12 | - | 1 | - | - | - | - |
|  | 13 | - | - | - | 1 | - | - |
| pgp4 | 1 | 20 | 62 | 9 | - | - | 34 |
|  | 2 | - | - | - | 22 | - | - |
|  | 4 | - | - | - | - | 10 | - |
| pgp5 | 1 | - | - | - | - | 5 | 31 |
|  | 2 | 19 | - | - | - | - | - |
|  | 3 | - | - | - | 21 | - | - |
|  | 4 | - | 62 | - | - | - | - |
|  | 5 | - | - | - | - | - | 2 |
|  | 6 | 1 | - | - | - | - | - |
|  | 7 | - | - | 1 | - | - | - |
|  | 8 | - | - | - | - | 5 | - |
|  | 9 | - | - | 5 | - | - | - |
|  | 10 | - | - | - | - | - | 1 |
|  | 11 | - | - | 2 | - | - | - |
|  | 15 | - | - | 1 | - | - | - |
|  | 16 | - | - | - | 1 | - | - |
| pgp6 | 1 | - | - | - | - | - | 28 |
|  | 2 | - | - | - | 22 | - | - |
|  | 3 | 17 | - | - | - | 10 | - |
|  | 4 | - | 58 | - | - | - | - |
|  | 6 | - | - | 1 | - | - | - |
|  | 7 | - | 1 | - | - | - | - |
|  | 8 | - | - | - | - | - | 1 |
|  | 9 | - | - | - | - | - | 1 |
|  | 10 | - | - | 8 | - | - | - |
|  | 11 | - | 1 | - | - | - | - |
|  | 12 | - | 1 | - | - | - | - |
|  | 14 | 1 | - | - | - | - | - |
|  | 15 | - | - | - | - | - | 3 |
|  | 16 | 1 | - | - | - | - | - |
|  | 18 | - | 1 | - | - | - | - |
|  | 19 | - | - | - | - | - | 1 |
|  | 22 | 1 | - | - | - | - | - |
| pgp7 | 1 | 14 | - | - | - | 10 | 32 |
|  | 2 | - | - | - | - | - | 1 |
|  | 4 | - | - | - | 19 | - | - |
|  | 5 | - | 61 | - | - | - | - |
|  | 6 | - | - | - | 3 | - | - |
|  | 7 | 1 | - | - | - | - | - |
|  | 8 | - | 1 | - | - | - | - |
|  | 9 | - | - | - | - | - | 1 |
|  | 10 | 3 | - | - | - | - | - |
|  | 12 | - | - | 6 | - | - | - |
|  | 13 | - | - | 3 | - | - | - |
|  | 15 | 1 | - | - | - | - | - |
|  | 16 | 1 | - | - | - | - | - |
| pgp8 | 1 | 17 | - | - | - | 9 | 34 |
|  | 2 | - | - | - | 22 | - | - |
|  | 3 | 1 | - | - | - | - | - |
|  | 4 | - | 38 | - | - | - | - |
|  | 5 | - | 24 | - | - | - | - |
|  | 8 | - | - | 3 | - | - | - |
|  | 9 | 1 | - | - | - | - | - |
|  | 10 | - | - | - | - | 1 | - |
|  | 11 | - | - | 5 | - | - | - |
|  | 12 | 1 | - | - | - | - | - |
|  | 18 | - | - | 1 | - | - | - |
|  | | | | | | | |
